# Impact of a risk based breast screening decision aid on understanding, acceptance and decision making

**DOI:** 10.1038/s41523-023-00569-4

**Published:** 2023-08-08

**Authors:** Jocelyn Lippey, Louise Keogh, Ian Campbell, Gregory Bruce Mann, Laura Elenor Forrest

**Affiliations:** 1 Sir Peter MacCallum Department of Oncology, Melbourne, Australia; 2 University of Melbourne, Department of Surgery, Melbourne, Australia; 3 St. Vincent’s Hospital, Department of Surgery, Fitzroy, Australia; 4 University of Melbourne, Melbourne School of Population and Global Health, Melbourne, Australia; 5 Cancer Genetics Laboratory, Peter MacCallum Cancer Centre, Melbourne, Australia; 6 Department of Surgery, The University of Melbourne, Melbourne, Australia; 7 Breast Service, The Royal Melbourne Hospital, Melbourne, Australia; 8 Parkville Familial Cancer Centre, Peter MacCallum Cancer Centre, Melbourne, Australia

**Keywords:** Population screening, Breast cancer

## Abstract

Internationally, population breast cancer screening is moving towards a risk-stratified approach and requires engagement and acceptance from current and future screening clients. A decision aid (www.defineau.org) was developed based on women’s views, values, and knowledge regarding risk-stratified breast cancer screening. This study aims to evaluate the impact of the decision aid on women’s knowledge, risk perception, acceptance of risk assessment and change of screening frequency, and decision-making. Here we report the results of a pre and post-survey in which women who are clients of BreastScreen Victoria were invited to complete an online questionnaire before and after viewing the decision aid. 3200 potential participants were invited, 242 responded with 127 participants completing both surveys. After reviewing the decision aid there was a significant change in knowledge, acceptance of risk-stratified breast cancer screening and of decreased frequency screening for lower risk. High levels of acceptance of risk stratification, genetic testing and broad support for tailored screening persisted pre and post review. The DEFINE decision aid has a positive impact on acceptance of lower frequency screening, a major barrier to the success of a risk-stratified program and may contribute to facilitating change to the population breast screening program in Australia.

## Introduction

Since the introduction of population-based breast cancer screening significant inroads have been made in the understanding of breast cancer risk^1^ particularly the role of factors such as mammographic density^2,3^ and genetic factors and their impact on an individual’s risk^4,5^. Potential harms of population screening such as overdiagnosis and false positives^6,7^ have come to light as well as significant advancements in screening technology. Despite these changes only small shifts such as the 2014 inclusion of the family history policy^8^ and breast density notification in some Australian states^9^, have altered to reflect this. Thirty years on, age and gender are still the predominant factors used to assess screening eligibility and breast cancer risk in the general population.

Risk-based screening, whereby recommendations are dependent on breast cancer risk category, the calculation of which would include mammographic density^10,11^, has the potential to further reduce breast cancer mortality by lowering rates of interval cancers in higher risk individuals^12,13^. Planning for the change of program structure is underway for breast cancer screening both internationally and in Australia^10,11,14,15^. Several international projects are underway in the UK^13,16,17^, Canada^18^, Europe^19^, Italy^20^ and USA^21,22^ to further understand the possible advantages of this approach including the role genomics may play in risk assessment.

Women who currently participate in routine population breast cancer screening are interested and supportive of a risk-stratified approach, including the use of genomics to assess risk^23,24^. However, there was less acceptance of decreased screening frequency if women are found to be lower than average risk^25,26^. Successful implementation of a risk-stratified approach to population breast cancer screening will depend on engagement and understanding from consumers, which will require comprehensive, and culturally sensitive communication made available in plain language.

A purpose built, online Decision aid (DA) called DEFINE (https://www.defineau.org) was developed incorporating current breast screening participants’ values, educational and decisional needs to support the process of choosing between current or risk-stratified screening^24,27^. An iterative process was undertaken with input from experts in a variety of fields as well as consumers who directed the content and improved readability.

The expansion in understanding of breast cancer risk, such as the impact of mammographic density and genetics, including polygenic risk information, has been shown to improve the assessment of an individual’s risk. Incorporating these factors into existing risk prediction models significantly improves risk prediction^28–30^; however, this benefit needs to be weighed up against feasibility and the increased expense and complexity of this personalised assessment especially collection of detailed family history. The inclusion of genetics requires careful consideration of the ethical, social, and psychological implications of genetic testing, and communication with consumers will play a critical role in managing these issues.

Decision making for an individual around screening is complex, personal, and likely to be based on emotion, experience or instinct rather than factual information^31^. There is an overwhelmingly positive opinion regarding the advantages of breast screening^32^ and people tend to overestimate benefits and underestimate harms in population based screening^33^. Informed decision making requires the provision of information covering both the benefits and harm of an intervention^34^ therefore, especially in the screening context where this has not always been the case, attention is needed to give education and communication with a balanced approach.

DAs, designed to assist people make health-related decisions that align with their personal values, can clarify decisional uncertainty, as well as weigh up the potential benefits and harms of a certain intervention. Overall, they improve understanding of clinical options, create more realistic expectations about outcomes and have been attributed to a higher likelihood of an informed value-based decision^35^. This has been a consistent finding throughout numerous studies of screening, including a meta-analysis on mammographic screening, confirming that DAs improve knowledge and promote informed decision making^36^. An online DA has the advantages over paper-based materials of accessibility, personalisation as well as being easily updated as information changes.^37^. An online DA has the potential to be a powerful tool towards informed decision making for risk-stratified breast cancer screening.

This paper reports on the impact of DEFINE on knowledge of breast cancer risk and risk-stratified screening, acceptability of different screening frequencies as well as informed decision making for women participating in the current breast screening program in Victoria, Australia.

## Results

### Participants

3200 email invitations were distributed, and 242 (8%) women completed the pre-DA review survey. 127 (53%) women completed both the pre and post-surveys. Demographic data is summarised in Table 1. Participants in this study were of higher average age and had an overrepresentation of higher Index of Relative Socio-economic Advantage and Disadvantage (IRSAD) postcodes compared to BreastScreen participants which are evenly distributed amongst these quintiles^38^. A summary of missing results is presented in Supplementary Table 1.

**Table 1:** Participant demographics.

	Respondents who completed pre-Questionnaire only *n* = 115 (%)	Respondents who completed both pre- and post-questionnaires *n* = 127 (%)	Total participants *n* = 242 (%)
Age	64 (range 43–74)	67 (range 51–74)	66 (range 40–74)
*Marital status*			
Partnered	89 (77%)	99 (78%)	188 (78%)
Not partnered or other	26 (23%)	28 (22%)	54 (22%)
*IRSAD*			
Lowest quintile	12 (10%)	12 (10%)	24 (10%)
Second quintile	25 (22%)	17 (13%)	42 (17%)
Third quintile	18 (16%)	27 (21%)	45 (19%)
Fourth quintile	21 (18%)	32 (25%)	53 (22%)
Highest quintile	39 (34%)	39 (31%)	78 (32%)
*Employment*			
Employed	49 (44%)	47 (38%)	93 (38%)
Not employed (incl. retired)	62 (56%)	77 (62%)	149 (62%)
*Education*			
University degree	77 (67%)	85 (67%)	162 (67%)
High school	20 (17%)	27 (21%)	47 (19%)
Did not complete high school	22 (19%)	11 (9%)	23 (10%)
Trade	2 (2%)	1 (1%)	3 (1%)
Missing	4 (3%)	3 (2%)	7 (3%)

### Breast cancer and screening experience

The participants were well-experienced BreastScreen attendees with 197 of the 241 (82%) participants who responded to the question (one missing response) having had more than three mammograms. From the initial cohort of 242 respondents, 29% had a prior breast biopsy and 22% reported a family history of breast cancer. Table 2 lists participants’ breast cancer and screening experience.

**Table 2:** Breast cancer and screening experience.

Mammogram experience	Respondents who completed pre-questionnaire only *n* = 115 (%)	Respondents who completed both pre- and post-questionnaires *n* = 127 (%)	Total participants *n* = 242 (%)
Never had	0 (0%)	0 (0%)	0 (0%)
1–3 prior mammograms	11 (10%)	12 (10%)	23 (10%)
>3 mammograms	93 (81%)	104 (82%)	197 (81%)
Unsure or missing	11 (10%)	11 (9%)	22 (9%)
*Biopsy experience*			
Have had breast biopsy	33 (27%)	36 (28%)	69 (29%)
Never had breast biopsy	78 (68%)	89 (70%)	167 (69%)
Unsure	3 (3%)	2 (2%)	5 (2%)
*Missing*	1 (1%)	0 (0%)	1 (0%)
*Breast cancer*			
Yes	1 (1%)	0 (0%)	1 (0%)
No	113 (98%)	126 (99%)	239 (99%)
Unsure	0 (0%)	1 (1%)	1 (0%)
*Missing*	1 (1%)	0 (0%)	1 (0%)
*Ovarian cancer*			
Yes	4 (3%)	1 (1%)	5 (2%)
No	108 (94%)	126 (99%)	234 (97%)
*Missing*	3 (3%)	0 (0%)	3 (1%)
*Family history—first degree*			
No cancers	43 (37%)	42 (33%)	85 (35%)
Breast cancer	19 (17%)	35 (28%)	54 (22%)
Ovarian cancer	8 (7%)	7 (6%)	15 (6%)
Other types of cancer	64 (56%)	61 (48%)	125 (52%)
*Missing*	0 (0%)	2 (2%)	2 (1%)
*Genetic testing (unspecified)*			
Yes	3 (3%)	7 (6%)	10 (4%)
No	112 (97%)	116 (91%)	228 (94%)
Unsure	0 (0%)	4 (3%)	4 (2%)

Family history response allowed for more than one type of cancer accounting for the total being >100%. Genetic testing was open to interpretation—no specification was given to specific genes tested.

### Risk perception

There was a normal distribution of responses on how likely a participant felt she was to develop breast cancer in her lifetime and no significant change between responses for pre and post DA review (scale from 0% “no chance of developing breast cancer” to 100% “will definitely develop breast cancer”—pre-review mean (*M*) = 41.0 (Standard deviation (SD) 22.1), post-review *M* = 44.1 (SD 22.6). The increase of scores indicates women perceived their breast cancer risk as slightly higher after reviewing the DA although this was not statistically significant (95% confidence interval (CI) −0.9 to 5.3, *P* value = 0.161).

### Breast cancer worry and overall anxiety

Breast cancer worry was normally distributed with 50% of all respondents (*n* = 63 with no missing responses) choosing the middle response of rarely worrying about breast cancer, 24% (*n* = 30) often or sometimes worrying, and 27% (n = 34) never worrying.

Overall anxiety, measured by OASIS, was low (*M* = 2.6, range 0–25, SD 3.0). There was a weakly positive association between a participant’s level of worry about developing breast cancer and their perceived risk of developing breast cancer (Spearman’s rho = 0.28 *P* value < 0.001).

### Knowledge of breast cancer risk factors

There was a significant increase in self-rated breast cancer risk factor knowledge pre (*M* = 65.5, SD = 20.0) compared to post (*M* = 75.4, SD = 16.8) reviewing the DA (*t* test 10.0; 95% CI 6.3–13.6; *P* < 0.001) and similarly for the risk factor knowledge test before (*M* = 4.6 SD = 2.0) and after (*M* = 6.0, SD = 2.0) (*t* test 1.4; 95% CI 1.0–1.7; *P* < 0.001). There was a weakly positive correlation between the post-test breast cancer risk factor knowledge test scores and self-reported knowledge on risk factors (Spearman’s rho = 0.3; *P* < 0.001) suggesting participants had high levels of insight into their understanding of breast cancer risk factors.

### Understanding of risk-stratified screening

**Fig. 1:**
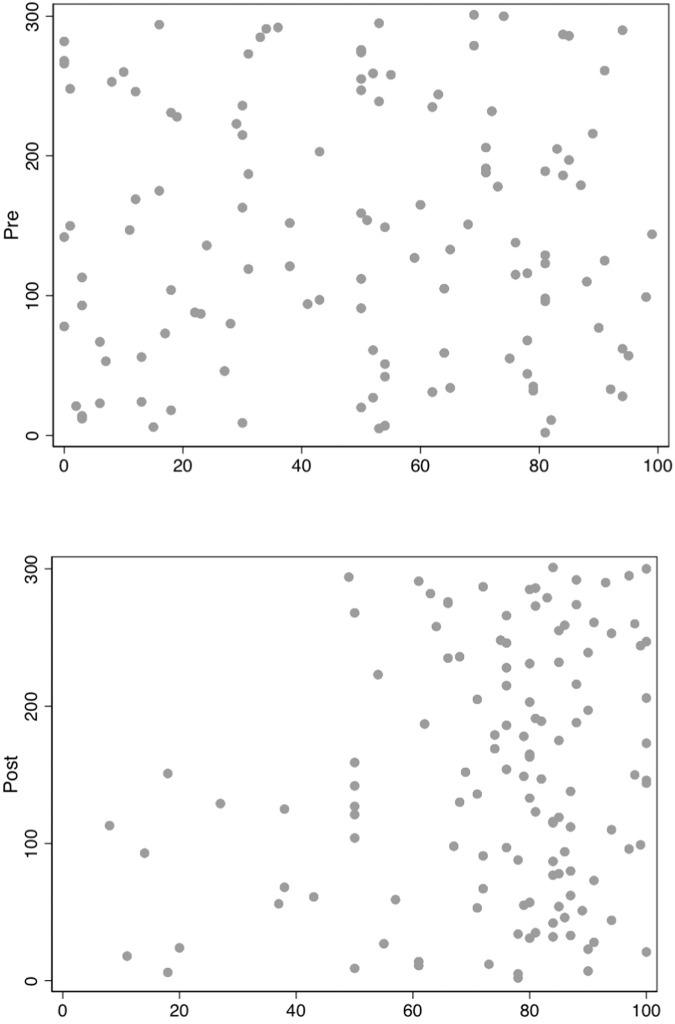
Quantitative self reported knowledge of personalised breast screening. Scatterplots of responses to “How do you rate your knowledge of individualised or personalised breast screening on a scale of 1 to 100 where 1 is poor and 100 is excellent?” prior to reviewing the website above and after reviewing the website below. The blue dots represent each participant’s response showing a substantial improvement in self-rated knowledge of individualised breast screening. *n* = 123 pre and 125 post (4 missing responses pre and 2 missing responses post).

Similarly, there was a statistically significant increase in how much women felt they knew about individualised breast screening with an increase mean score from 49.0 (SD = 29) to 74.4 (SD = 20.4, range 0–100) post reviewing the DA (95% CI 19.5–30.8, *P* < 0.001). Figure 1 shows the improvement of subjective rating of risk-stratified screening of the participants by contrasting the distribution of responses prior and after reviewing the website in a scatterplot.

**Fig. 2:**
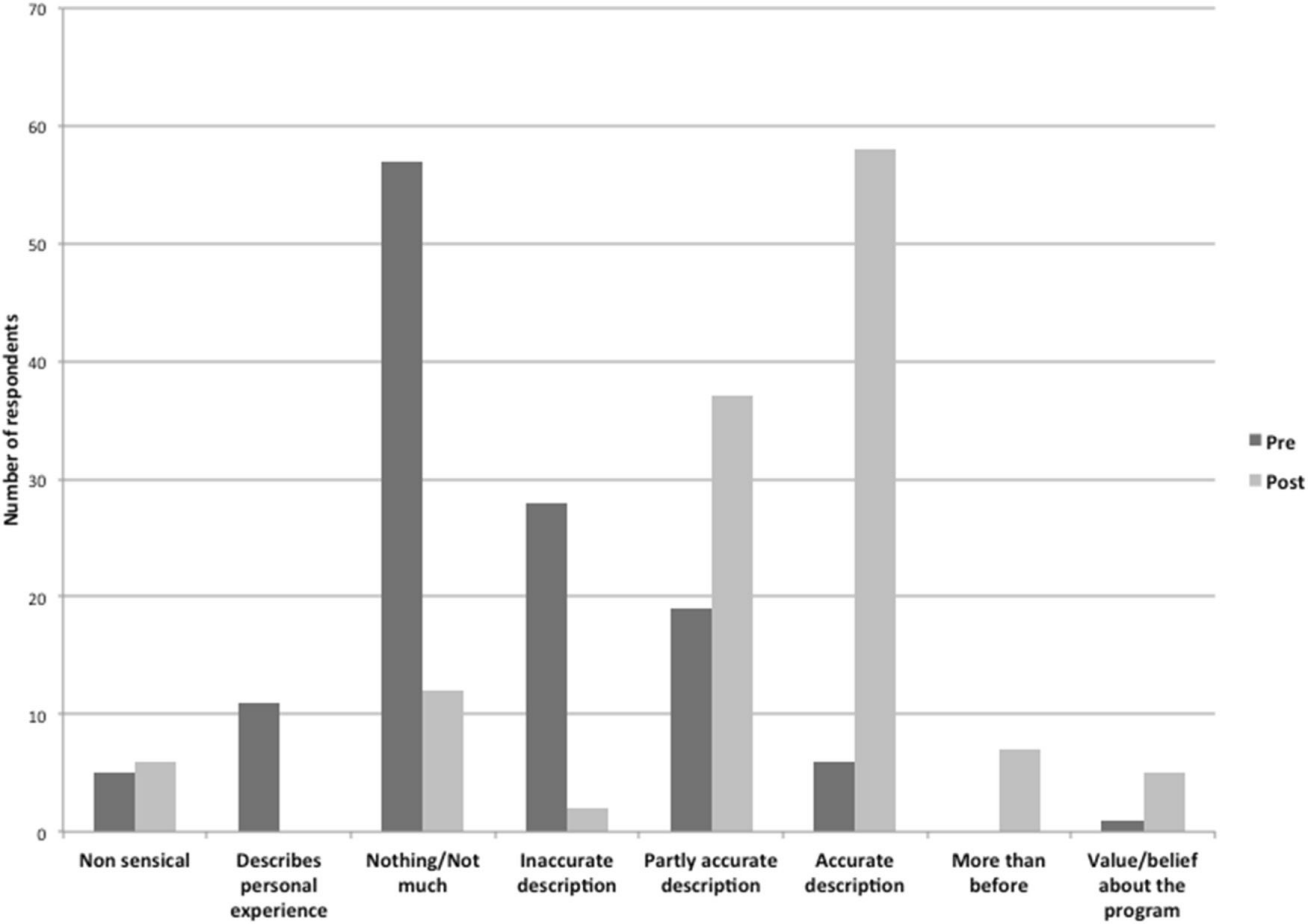
Qualitative responses to knowledge of personalised breast screening. Open coding response frequencies pre and post DA review to “What do you know about individualised or personalised breast cancer screening?”. *n* = 127 Figure 2 legend: (a) pre, (b) post.

Open coding to assess the accuracy of responses to the free text question, “What do you know about individualised or personalised breast cancer screening?”., revealed eight categories. See Table 3 for categories and response examples. Given this was a compulsory question, five women in the pre and six women in the post-survey responded with non-sensical responses. There was a marked shift towards a more accurate response in the description of personalised screening after reviewing the website, as shown in Fig. 2.

**Table 3:** Themes and examples for open coding to “What do you know about individualised or personalised breast cancer screening?”.

Theme	Example
Nonsensical	“mm”, “4”
Describes personal screening experience	“Because my mother has had two bouts of breast cancer I am very particular about having my tests done”
Nothing/not much/unsure	“nil”
Inaccurate description	“I know the mammogram is checked and hopefully, if any cancer present it will be picked up but I know this is not always the case.”
Partly accurate description	“Higher risks, more frequent screens”
Accurate description	“I think it means that instead of a blanket approach where everyone gets a mammogram over a certain age that instead it depends on your risk factors”
More than before	“Only what I have just read”
Value or belief	“Sounds like a good idea but I would still like the ‘insurance’ of a 2 year mammogram” “Enough to be interested”

### Interest in risk-stratified screening overall

**Fig. 3:**
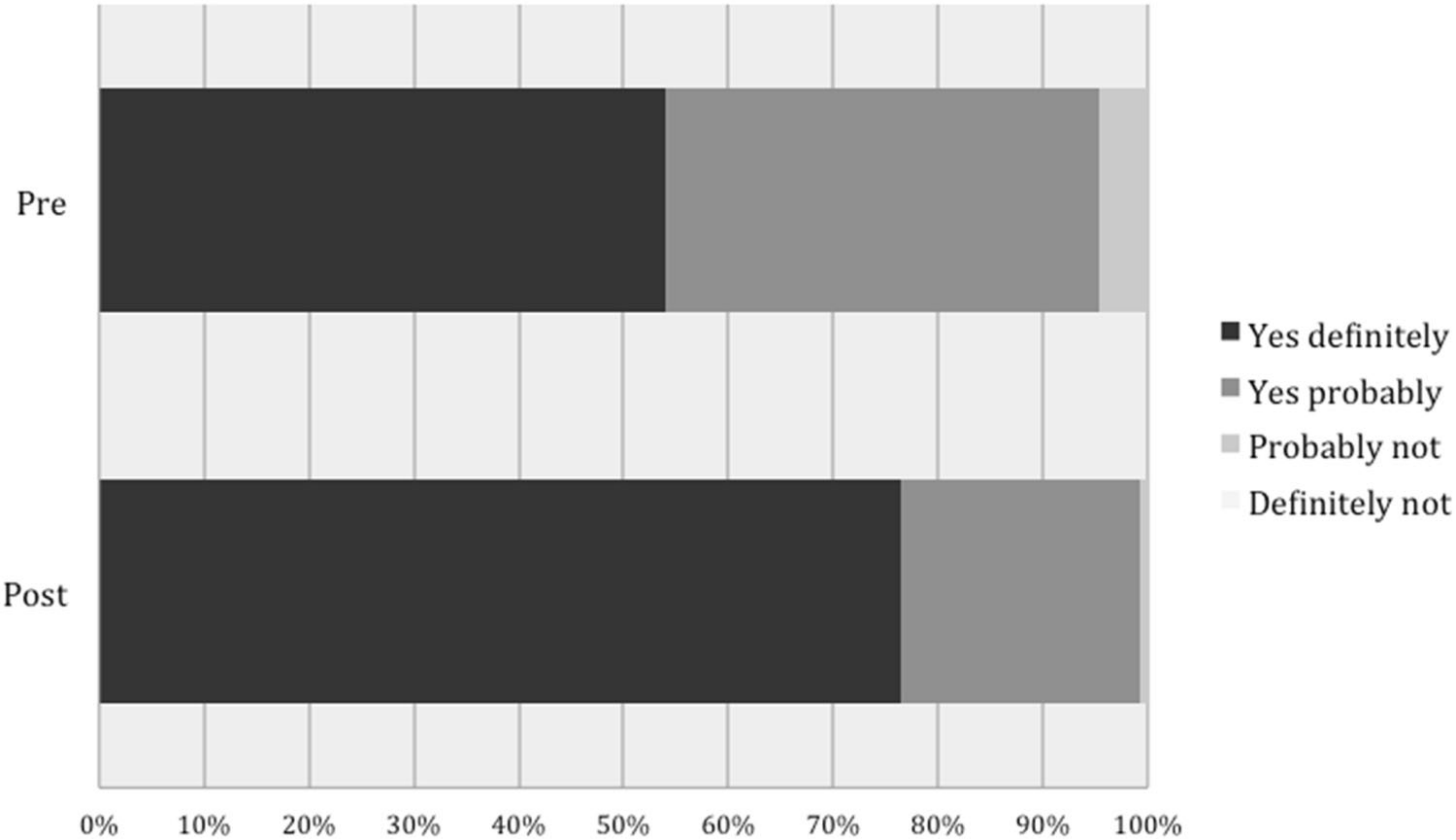
Individualised screening interest. Responses to “Would you be interested in breast screening if each woman was managed differently depending on her personal breast cancer risk?”. *n* = 127 Figure 3 legend (a) pre, (b) post, (i) Yes definitely, (ii) Yes probably, (iii) Probably not, (iv) Definitely not.

Initial levels of interest in risk-stratified screening were very high. Of the 241 respondents who answered the question to the survey pre-DA review, 226 participants (93.7%) were definitely or probably interested in a risk-stratified approach to breast cancer screening. Analysis of the 127 participants who completed both pre and post-surveys saw a shift from 122 to 126 women into either the “Yes probably” or “Yes definitely” group, illustrated in Fig. 3. Grouping of responses into binary outcomes (“Yes probably” and “Yes definitely” into Interested and “Probably not” and “Definitely not” into Not interested) demonstrated no statistically significant change (*P* = 0.063).

There was a slightly higher rate of interest in the women who completed both pre and post DA review questionnaires compared to the 115 participants who did not complete the survey for after DA review, however this was not statistically significant (*P* = 0.779).

There was no association between levels of breast cancer worry and interest in risk-stratified breast screening (Spearman’s rho = −0.159, *P* = 0.074) and similarly, there was no association between women’s levels on anxiety as measure on the OASIS scale and their interest in risk-stratified breast screening (Spearman’s rho = −0.084, *P* = 0.347).

### Acceptability of varying screening intervals

**Fig. 4:**
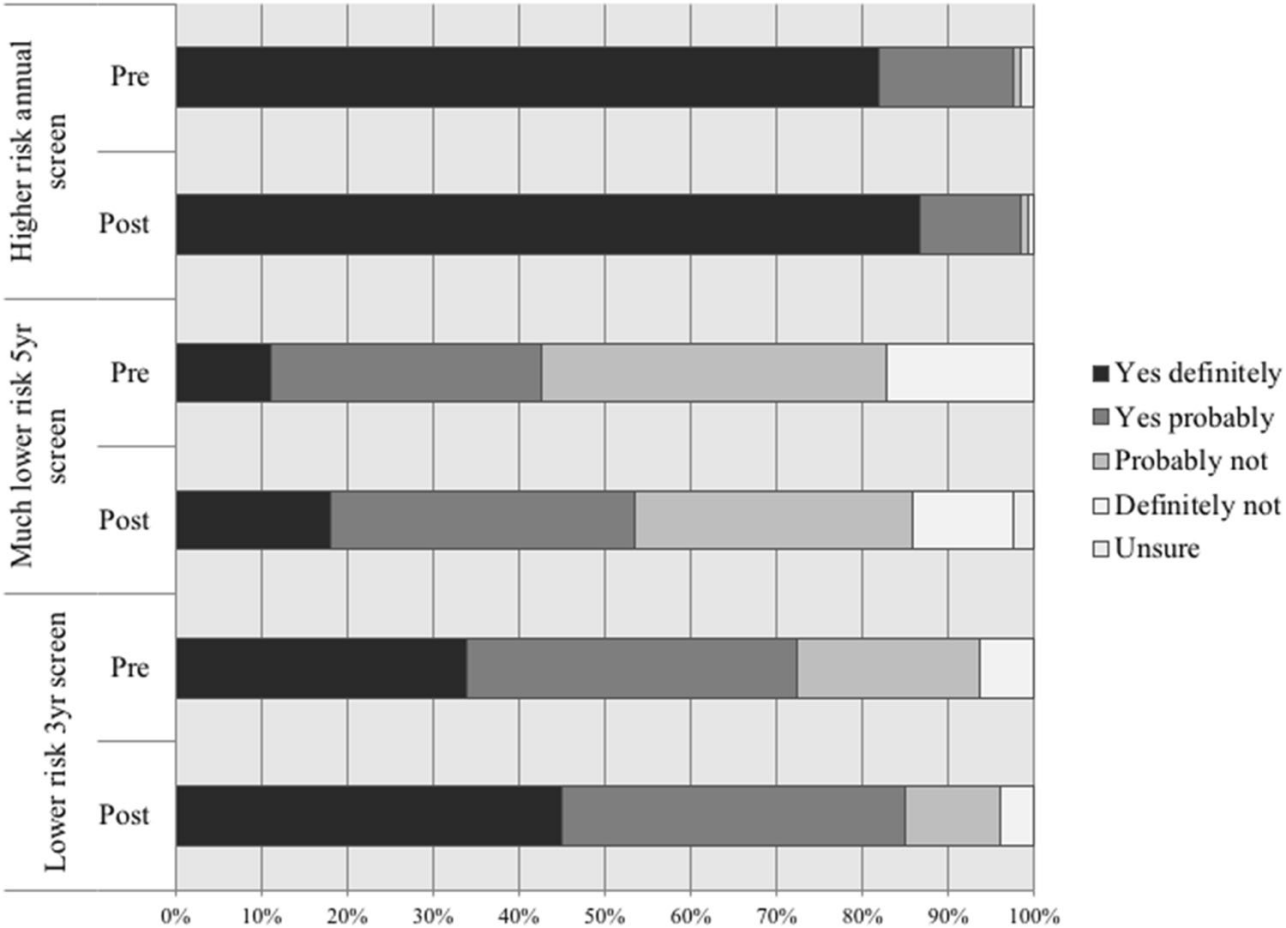
Responses to acceptance of different screening frequencies depending on level of risk. *n* = 127 Figure 4 legend: (a) pre, (b) post, (1) Higher risk annual screen, (2) Much lower risk 5 year screen, (3) Lower risk 3 year screen, (i) Yes definitely, (ii) Yes probably, (iii) Probably not, (iv) Definitely not, (v) Unsure.

In the pre-DA survey, there were very high levels of acceptance for having an annual mammogram if high risk (124 of the 125 respondents who answered the question, 99%) compared to the acceptance of having a 3-year screening interval if deemed lower than average risk (92 respondents with no missing responses, 72%). Figure 4 outlines the responses to all three questions of varying screening intervals. Of note, 35 women (28%) would not be accepting of a 3-year screening interval if lower than average risk prior to reviewing the DA but this reduced to 19 (15%) after reviewing the DA. Grouping the responses into binary categories resulted in a statistically significant shift towards acceptance of a 3-year screening interval if deemed lower than average risk (*P* = 0.005).

Levels of acceptance for annual mammograms if higher than average risk was exceptionally high with only one of the 125 respondents who answered the question (0.8%) not interested prior to reviewing the DA and two of the 127 respondents (1.6%) in the post review survey. On binary analysis there was no significant change between the two responses (*P* = 0.564).

In terms of a 5-year screening interval of much lower than average risk, this was acceptable to only 43% of women (54 of 127 respondents) pre-DA review, however this increased 54% (68 of 127 respondents) after reviewing the website. Grouping the outcomes for acceptance of a 5-year interval into binary categories there was a statistically significant shift towards acceptance (*P* = 0.004).

There was no relationship between breast cancer worry and accepting a five year interval for screening if lower risk (Spearman’s rho = −0.1, *P* = 0.343).

### Acceptability of genetic testing incorporated into screening and risk assessment

**Fig. 5:**
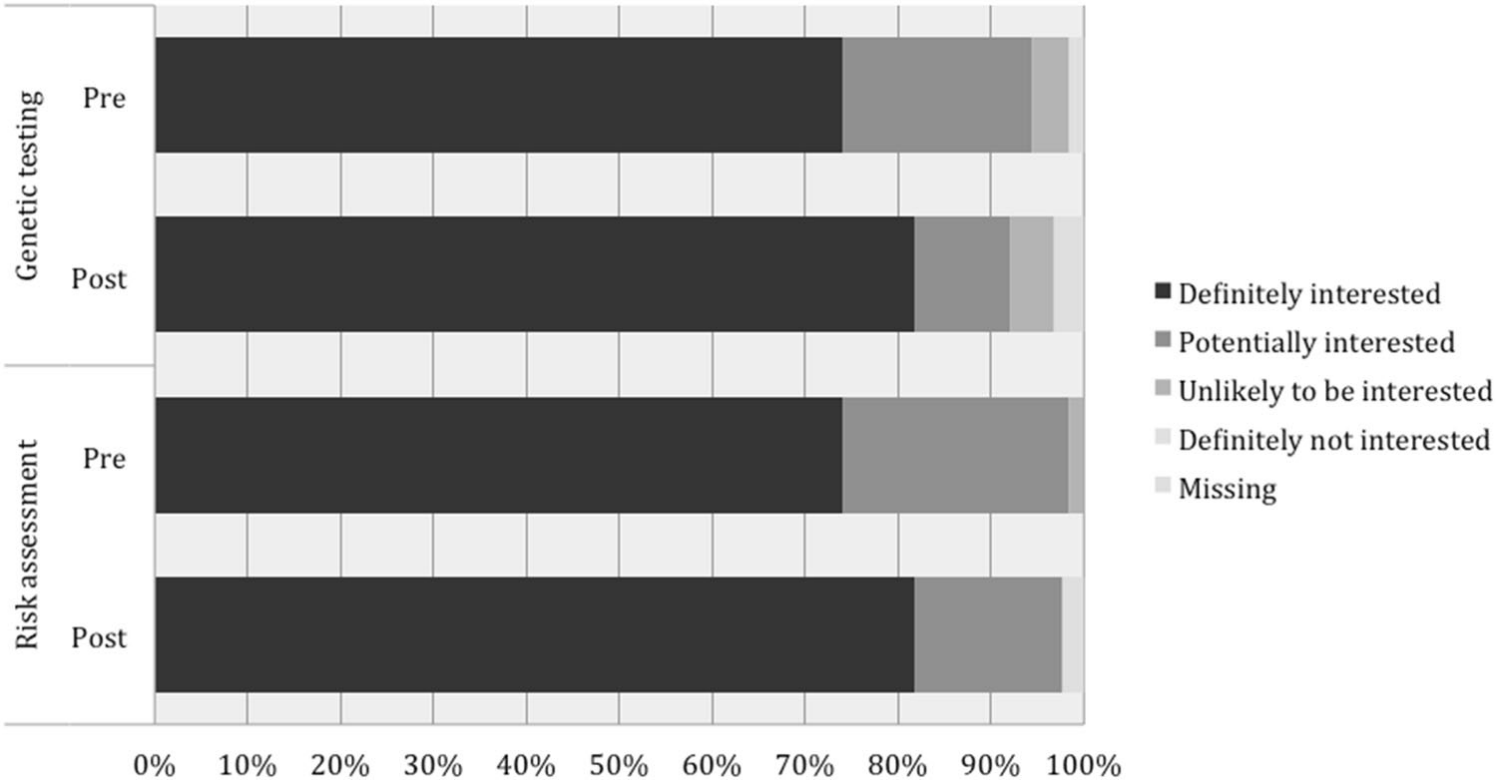
Responses to acceptance if risk assessment and genetic testing being incorporated into routine BreastScreen practice. *n* = 127 Figure 5 legend: (a) pre (b) post, (1) Risk assessment, (2) Genetic testing, (i) Definitely interested, (ii) Potentially interested, (iii) Unlikely to be interested, (iv) Definitely not interested, (v) Missing.

Levels of acceptance of genetic testing were very high with 120 of the 126 respondents who answered the question (95.2%) interested in the pre-DA survey and 117 of the 123 participants who answered the question (95.1%) interested in the post-DA survey. Similarly, in regard to accepting a risk assessment, this was very high in the pre-DA survey with 125 participants (98.4%) accepting this before and 124 (100%—3 missing responses) in the post-DA survey. This compares to 95% (109 of 115) of respondents who did not complete the post-DA survey who were accepting of a risk assessment. Figure 5 demonstrates responses to these questions.

### Informed decision making

95 women (75% of respondents) gave adequate responses to the free text response describing risk-stratified screening after reviewing the DA indicating adequate knowledge. 64 of those women had positive values and preferred individualised screening and three participants had negative values and preferred the current program. 67 participants or 53% of the cohort have been classified as making an informed decision. Seven participants were unsure which option they prefer and 20 did not respond to the question of preference.

### Satisfaction with decision

Satisfaction with decision was overall very high with 113 of the 125 respondents who answered the question (90%) rating a 0–6 high score. The remaining 12 respondents (10%) were in the intermediate 7–12 range and no participants had low levels of decision satisfaction.

### Website feedback

Responses to specific questions around utility and usefulness of the website are summarised in Table 4. Interestingly, 114 (90%) of women spent 30 min or less and participants reported very little anxiety while reviewing the website. Most respondents reported the website content was new to them with 91 respondents (73%) reporting some of the information was new to them compared to 7 (6%) reporting none of it was new.

**Table 4:** Website feedback questions and responses.

Question	Response option	*n* = 127 (%)
Time spent on website	0–10 min	42 (34%)
	10–30 min	72 (58%)
	30–60 min	11 (9%)
	>60 mins	–
How much of the information was new to you?	All	1 (1%)
	Most	26 (21%)
	Some	91 (73%)
	None	7 (6%)
How would you describe the website in terms of ease of understanding?	Excellent	52 (42%)
	Very good	46 (37%)
	Good	22 (18%)
	Moderate	5 (4%)
	Poor	–
Did you click on any of the links to other references/articles/website	Yes	55 (44%)
	No	62 (50%)
	Unsure	7 (6%)
Did you click on any of the words on the website for an explanation/definition of a medical term?	Yes	22 (18%)
	No	104 (83%)
How helpful was the website in explaining your personal risk of breast cancer?	Excellent	32 (26%)
	Very good	54 (43%)
	Good	29 (23%)
	Moderate	7 (6%)
	Poor	3 (2%)
How would you describe how useful the website was helping you decide if individualised breast cancer screening was right for you?	Excellent	40 (32%)
	Very good	53 (42%)
	Good	25 (20%)
	Moderate	7 (6%)
	Poor	–
I felt anxious or worried reading the website	All of the time	1 (1%)
	Some of the time	7 (6%)
	Once or twice	14 (11%)
	Not at all	91 (72%)
	Unanswered	14 (11%)
Was there anything which confused you?	Yes	8 (7%)
	No	116 (94%)
Do you have any suggestions as to how the website could be improved?	Unanswered	55 (43%)
No	35 (28%)	
Technical issues with functionality or internet connection	7 (10%)	
Feedback on wording of survey questions	5 (7%)	
Concerned about genetic testing and safety of personal data	2 (3%)	
Requesting support for non-English speakers	2 (3%)	
Wanted more information about screening after the age of 74	2 (3%)	
Wanted percentages represented as one in x	1 (1%)	
Specific questions about risk factors	1 (1%)	
Wanted a personalised risk assessment	1 (1%)	
Wanted more information about the risks associated with having mammograms	1 (1%)	
Repetitive	1 (1%)	
Requests simpler language to be used	1 (1%)	

## Discussion

Our findings confirm that the DEFINE online DA (www.defineau.org) improves knowledge and in principle acceptance of a risk-stratified breast screening approach as well as allowing for informed decision-making. These results are in keeping with assessments of other DAs which consistently report significant improvements in knowledge^39–47^ and were confirmed in a Cochrane review of 105 studies reporting on DAs which have a significant impact on knowledge^48^.

In terms of acceptance of risk-stratified breast cancer screening, previous work has consistently reported strong levels of acceptance for risk assessment and a risk-stratified approach but a persisting reticence for accepting lower frequency screens for women who are deemed lower than average risk^23,25,26,49–52^. A purpose-built DA could manage these hesitancies, and our work demonstrates that education plays a critical role in theoretical acceptance of less frequent screening.

While other studies have shown that a participant’s perceived high risk influences acceptance of more frequent screening based on objective risk assessment^26^, this was not observed in our study. This discrepancy might be explained by the fact that the majority of our participants felt themselves at exceptionally high level of risk of developing breast cancer with a median perceived lifetime risk of developing breast cancer of 41%.

The reluctance to accept lower frequency screenings if lower than average risk is understandable from a cohort of women who have been advised for two-yearly screening and they have been adherent to this for many years. 82% of our cohort had more than three prior mammograms and only a small group of women (12 participants) had 1–3 prior mammograms. Given the disparate group sizes between less and more prior screening rounds, meaningful differences could not be ascertained in terms of acceptability of differing screening frequencies, or risk stratification more broadly. It is plausible a group of women with less experience in the current system would have different attitudes towards a lower frequency of screening and this would be an interesting future study.

The implications of our work for risk-stratified breast screening into an Australian context is reassuring in that we demonstrate women currently participating would not be dissuaded by a change in the program. We show women participating in our current program are interested in and motivated by this approach. There is broad acceptance of having risk assessed within our existing system as well as in the inclusion of genetic testing to this risk calculation. We have previously reported a proportion of current BreastScreen clients may have concerns about data security and privacy issues associated with genetic testing^24^; however, our current results quantify those concerns to be small.

The first limitation of this study is the skewed sample of women which were recruited. By recruiting women already actively involved in population breast screening it is difficult to generalise these findings to the broader population including those women who have little or no experience with our current breast screening program. Our cohort is not a representative sample of the broader BreastScreen community given they were highly experienced with over 80% having had at least 3 prior mammograms and with an average age older than the average BreastScreen Victorian client. The impact of this experience and older age is challenging to quantify but is foreseeable as a confounding factor in risk perception and acceptability.

Secondly, with only an 8% uptake rate from the initial email invitation and an overrepresentation of higher socioeconomic postcodes^38^ and an higher age than the average screening population, we may not have a representative sample of the overall screening population in Victoria. The other indicator we may have sampled a very well-informed group was that 73% of the cohort reported only ‘some’ of the website information was new to them. The use of email for recruitment will also have sub-select participants with higher technological literacy which potentially biased our cohort. This method of recruitment was selected as it most closely aligned with our tool being web based; however, a paper or in person-based recruitment strategy may have uncovered different results.

Thirdly, we note just over half the participants completed both the pre and post-questionnaires. The group that completed only the pre-DA questionnaire was slightly younger with a marginally lower interest in risk-stratified screening and this further may limit the generalisability of our findings.

Another limitation is the hypothetical nature of the responses. There may be disparity between intention and behaviour and women in this cohort were not committing to an actual change or acceptance, rather indicating potential intentions to an approach not yet available to them.

We have custom built an effective decision aid which can support women’s decision making around a risk-stratified model and have demonstrated that even brief intervention significantly improved understanding and acceptability of a risk-stratified approach to population breast screening in Australia.

International work from Canada, UK and Europe has consistently shown women are hesitant about reducing their screening interval^23,25,26,49–51^ but we have demonstrated this hesitancy can be lessened with brief educational and values assessment intervention; however, future work is needed assess whether this translates into real-world behaviour change with higher levels of uptake as well as the impact on a screening naïve population.

The transition from our current one size fits all population breast screening model to a tailored or personalised approach is well underway^10,14^. We recognise the importance of patient involvement, education, and communication in a new population health intervention. Future directions for this DA are planned addressing key stake holders perspectives on the implementation of DAs into a risk-stratified breast screening approach in Australia.

## Methods

### Design

Single arm mixed methods survey conducted pre and post-review of an online DA, DEFINE.

### Decision aid development

The DA content was directed by results from focus groups conducted with participants who all attend BreastScreen Victoria^24^. Stepwise, iterative development of the online DA was guided by recommendations from the International Patient Decision Aid Standards (IPDAS) Collaboration^53,54^ and has been described previously^27^. DEFINE can be viewed at https://www.defineau.org and fulfills 26 of the 41 criteria on the IDPASi v3 criteria^53^.

### Participants and recruitment

Women currently participating in the BreastScreen Victoria (BSV) program who had a negative screen in the prior 6 months were invited via email to participate in the study. Through electoral roll data (voting in Australia is compulsory), BSV actively recruits women aged 50–74 years of age with an invitation letter at the age of 50. Women are eligible to participate from the age of 40 but are not actively invited by letter. Inclusion criteria for BSV is having identified as female on the electoral roll and being over the age of 50; however, there are clients within BSV outside of the target age range of 50–74. Women with breast cancer come out of the BSV program at diagnosis but are able to reenter at 5 years post-diagnosis. For this reason, women who had a prior breast cancer diagnosis were not excluded in this study in an attempt to represent the greater BSV population accurately.

Participants were randomly selected from the BSV database for a range of postcodes and ages. The invitation email included a link to the online pre-DA review survey. This initial survey ended with the link to DEFINE. After submitting the pre-review survey, an automated email was sent to the participant containing the post-DA review survey, allowing individual responses to be paired. At the start of the post-review website survey was a compulsory question asking “Have you reviewed the DEFINE website?”. Participants could not start the post-DA review questionnaire without ticking ‘yes’ to this question.

### Ethics approval and consent to participate

Ethics approval was obtained through Peter MacCallum Cancer Centre Ethics Committee (EC00235) Project number 17/194L. All procedures performed were in accordance with the ethical standards of the institutional research committee and with the 1964 Helsinki Declaration and its later amendments or comparable ethical standards.

All participants, including consumers, signed written informed consent forms approved by the ethics committee

### Questionnaire Measures

The study team constructed the mixed methods questionnaire as an iterative process. Where possible validated tools were used or existing tools modified for purpose. Table 5 outlines the survey domains, demonstrating which questions were repeated after a review of the website allowing for a direct comparison of responses.

**Table 5:** Questionnaire domains.

Pre	Post
*Demographic data*	
Risk factor knowledge/confidence ➨	Risk factor knowledge/confidence
Breast cancer worry and general anxiety	
Breast cancer risk perception ➨	Breast cancer risk perception
Intention to participate ➨	Intention to participate
Acceptability of risk assessment ➨	Acceptability of risk assessment
Acceptability of change of screening ➨	Acceptability of change of screening
	Attitudes/Values (Informed decision making)
	Satisfaction with decision scale
	Website feedback
	Satisfaction with decision aid

Demographic characteristics: Measured were: age, postcode (surrogate for socio-economic status), level of education, language spoken at home, experience with mammograms, prior breast biopsy, personal history of breast cancer and family history of breast cancer. Genetic testing was inquired about without specifying which genes had been tested.

Breast cancer risk factors knowledge: A knowledge measure of breast cancer risk factors was purpose-built using eight known breast cancer risk factors. Participants were required to “tick” as many of the factors they knew, and direct pre-post comparison was performed. The factors listed were – Mammographic density, family history, alcohol intake, body mass index, age, age of first pregnancy, age at menopause and being nulliparous^1^. Incorrect distractors (such as stress or the use of antiperspirant deodorants) were excluded to reduce the chance of confirmation bias reinforcing myths about breast cancer risk factors. Self-reflection of knowledge was assessed by a single question where participants rated their knowledge of breast cancer risk factors on a scale of 1–100 where 1 is poor and 100 is excellent.

Risk-stratified Breast screening knowledge: The only compulsory answer within the survey was a free text response to the question “What do you know about “individualised” or “personalised” breast screening?”. The free text responses were interrogated using a structured qualitative content analysis approach. Discrete codes, ‘accurate description’, ‘partially accurate’ or ‘value/belief about the program’, were defined and responses were compared pre and post for a change in understanding.

Self-reported knowledge of risk-stratified breast screening was quantified and compared with a question asking participates to self-rate their knowledge of risk-stratified screening where 1 is poor and 100 is excellent.

Breast cancer worry and general anxiety: A single question was posed to assess overall worry about developing breast cancer “How much do you worry about getting breast cancer?” with the potential responses being ‘never, rarely, sometimes, often, or most of the time’ which was adapted from the UK Health Information National Trends Survey^55^.

General anxiety was measured using the Overall Anxiety Severity and Impairment Scale (OASIS)^56^. This five-item tool measures each response using a 5-point Likert scale with questions being equally weighted to give a total score of 0–25. A score of 0–5 indicates mild or no anxiety, 6–10 indicates moderate, 11–15 severe and a score of 16–25 indicates extreme anxiety. We were interested in examining the role anxiety plays in decision-making^57^, and although anxiety is only one of many factors which may impair decision making we chose to focus on this as it has been documented to have a particularly pertinent role in breast cancer screening decisions^58^.

Breast cancer risk perception: Risk perception was assessed using three validated questions using numeric, verbal comparative scales adapted from Gurmankin Levy et al.^59^. Risk perception can be a strong motivator for uptake of health behaviours such as cancer screening^60^.

Intention to participate: Two questions assessed intention to participate in a risk-stratified breast screening program. The first being: “*If BreastScreen offered you the chance to take part in individualised breast screening in place of the current BreastScreen program, would you be:*” of which the response options were: definitely interested, potentially interested, unlikely to be interested, or definitely not interested. The second question “*Would you be interested in breast screening if each woman was managed differently depending on her personal breast cancer risk*?” had potential responses of: yes definitely, yes probably, probably not, definitely not.

Acceptability of risk assessment and genetic testing: Two questions examining acceptability of risk assessment as well as acceptance of genetic testing were asked: “*If you were offered an assessment of your personal breast cancer risk by BreastScreen at your next mammogram, would you be*” and “*If calculating your personal breast cancer risk meant having a blood test to look at your genes, would you be*” with available responses on a four-point Likert scale: definitely interested, potentially interested, unlikely to be interested and definitely not interested.

Acceptability of change of screening: Three questions addressed the issue of acceptance of differing screening frequencies (for example, mammograms every one or three years dependent on level of risk). Prior to these questions, there was a descriptor explaining the current Australian recommendations for biennial mammography. These purpose built questions were aimed at assessing the acceptability of individualised screening and the impact of the DA on level of acceptability. The first question was “*Hypothetically, if your personal breast cancer risk was lower than the average woman you could be advised to have a mammogram every 3 years. Would you be happy to have screening every 3 years if you were found to be low risk?”*. Available responses included yes definitely, yes probably, probably not, or definitely not.

The other two scenarios were gauging acceptance of a mammogram every 5 years if their risk were much lower than average and to have an annual mammogram if they were found to be higher risk.

Informed decision making: The multidimensional measure of informed choice (MMIC) acknowledges informed decision-making requires adequate knowledge, and alignment with values/attitudes and intention. This has been validated in the context of antenatal screening for Down’s syndrome^54,61,62^. In order to measure informed decision making we assessed three separate outcomes and combined three outputs of the post-website review survey: knowledge of risk-stratified screening from the free text response, attitudes of risk-stratified screening modified from the validated 6 questions developed by Dormandy^63^, and preference for current or risk-stratified screening. Participants were considered to have made an informed decision when they had adequate knowledge, positive values, and preferred risk-stratified screening or if they had adequate knowledge, negative values and preferred the current screening model. Adequate knowledge was defined as a response in the post-website review questionnaire to the open-ended question to “What do you know about “individualised” or “personalised” breast screening?” with ‘accurate description’ and ‘partly accurate description’ responses defined as adequate knowledge.

Satisfaction with decision-making: Satisfaction with decision scale is a validated six item scale modified from O’Connor’s decisional conflict scale^64^ evaluating satisfaction of a decision made. This measure was validated with a population of women deciding whether to take hormone replacement therapy^65^.

Each question is answered on a five-point Likert scale of strongly agree, agree, neither agree or disagree, disagree, or strongly disagree. Scores were totalled and categorised into high, moderate, or low satisfaction.

Website feedback and satisfaction: Nine questions assessing length of time spent reading website, clarity of information, ease of navigation and use of extra links embedded into text on the website which provided additional references.

Seven questions addressing responses to the website including emotional response and helpfulness of the website. The final question to the survey was open text “Do you have any suggestions as to how the website could be improved?”.

### Data collection

Study data were collected between February and July 2020 and managed using REDCap electronic data capture tools hosted at The University of Melbourne^66,67^.

### Data analysis

Categorical data were analysed with descriptive statistics. Pre and post-questionnaire data were analysed using a paired t test for continuous variables and Fisher’s exact test for categorical variables. Binary comparison assessing a pre-post review change in interest in risk-stratified screening and acceptance of differing screening frequencies was performed using a McNemar’s test. Comparisons were only performed using matched pre-post data and *t* tests were two-tailed. Correlation between anxiety, risk perception and acceptability was performed using Spearman’s rank-order correlation. Statistical analysis was performed using STATA^IC16^.

Content analysis for the free text response question regarding knowledge of risk-stratified screening was conducted initially by JL using an inductive approach coding into categories. These were co-coded by LF and where discrepancies arose, these were resolved by discussion between study investigators.

### Reporting summary

Further information on research design is available in the Nature Research Reporting Summary linked to this article.

### Supplementary information

Supplementary table 1

Reporting summary

## Data Availability

Can be accessed by contacting the corresponding author

## References

[1] "CancerAustralia". *Risk Factors for Breast Cancer: A Review of The Evidence* (Cancer Australia, 2018).

[2] Boyd NF, Martin LJ, Yaffe MJ, Minkin S (2011). Mammographic density and breast cancer risk: current understanding and future prospects. Breast Cancer Res..

[3] Boyd, N. F. *Mammographic Density and Risk of Breast Cancer* e57–e62 (ASCO, 2013). 10.14694/EdBook_AM.2013.33.e57 23714456

[4] Sawyer S (2012). A role for common genomic variants in the assessment of familial breast cancer. J. Clin. Oncol..

[5] Michailidou K (2015). Genome-wide association analysis of more than 120,000 individuals identifies 15 new susceptibility loci for breast cancer. Nat. Genet..

[6] Barratt A, Howard K, Irwig L, Salkeld G, Houssami N (2005). Model of outcomes of screening mammography: information to support informed choices. BMJ.

[7] Marmot MGAD, Cameron DA, Dewar JA, Thompson SG, Wilcox M (2012). The benefits and harms of breast cancer screening: an independent review. Lancet.

[8] BreastScreenVictoria. Family history of breast cancer and screening: BreastScreen Victoria (2017). Available from https://www.breastscreen.org.au/assets/resources/BSV-Family-history-of-breast-cancer-and-Screening.pdf.

[9] Dench EK (2020). Confusion and anxiety following breast density notification: fact or fiction?. J. Clin. Med..

[10] Allweis TM, Hermann N (2021). ASO author reflections: will breast cancer screening become personalized?. Ann. Surg. Oncol..

[11] Allweis TM, Hermann N, Berenstein-Molho R, Guindy M (2021). Personalized screening for breast cancer: rationale, present practices, and future directions. Ann. Surg. Oncol..

[12] Howell A (2014). Risk determination and prevention of breast cancer. Breast Cancer Res..

[13] Onega T (2014). Breast cancer screening in an era of personalized regimens: a conceptual model and National Cancer Institute initiative for risk-based and preference-based approaches at a population level. Cancer.

[14] Nickson C, Velentzis LS, Brennan P, Mann GB, Houssami N (2019). Improving breast cancer screening in Australia: a public health perspective. Public Health Res. Pract..

[15] Clift AK (2022). The current status of risk-stratified breast screening. Br. J. Cancer.

[16] Evans DG (2016). Improvement in risk prediction, early detection and prevention of breast cancer in the NHS Breast Screening Programme and family history clinics: a dual cohort study. Program. Grants Appl. Res..

[17] French DP (2020). What are the benefits and harms of risk stratified screening as part of the NHS breast screening Programme? Study protocol for a multi-site non-randomised comparison of BC-predict versus usual screening (NCT04359420). BMC Cancer.

[18] Brooks JD (2021). Personalized risk assessment for prevention and early detection of breast cancer: integration and implementation (PERSPECTIVE I&I). J. Personalized Med..

[19] Unicancer. MyPeBS [MyPeBS is an international EU-funded clinical study that evaluates a new breast cancer screening strategy]. Available from https://mypebs.eu. (2022).

[20] Paci E, Mantellini P, Giorgi Rossi P, Falini P, Puliti D (2013). Tailored Breast Screening Trial (TBST). Epidemiologia Prev..

[21] Eklund M (2019). The WISDOM personalized breast cancer screening trial: simulation study to assess potential bias and analytic approaches. JNCI Cancer Spectr..

[22] Esserman LJ, Study W, Athena I (2017). The WISDOM Study: breaking the deadlock in the breast cancer screening debate. NPJ Breast Cancer.

[23] Wheeler JCW (2022). Heterogeneity in how women value risk-stratified breast screening. Genet. Med..

[24] Lippey, J., Keogh, L. A., Mann, G. B., Campbell, I. G. & Forrest, L.E. “A natural progression”—Australian women’s attitudes about an individualised breast screening model. *Cancer Prevention Res*. **12**, canprevres.0443.2018 (2019) 10.1158/1940-6207.CAPR-18-0443 31003994

[25] Mbuya Bienge C (2021). Women’s views on multifactorial breast cancer risk assessment and risk-stratified screening: a population-based survey from four provinces in Canada. J. Personalized Med..

[26] Meisel SF (2015). Adjusting the frequency of mammography screening on the basis of genetic risk: attitudes among women in the UK. Breast.

[27] Lippey J, Keogh L, Campbell I, Mann GB, Forrest L (2022). Development and pilot testing of an online decision aid for women considering risk-stratified breast screening. J. Community Genet..

[28] van Veen EM (2018). Use of single-nucleotide polymorphisms and mammographic density plus classic risk factors for breast cancer risk prediction. JAMA Oncol..

[29] Brentnall AR (2015). Mammographic density adds accuracy to both the Tyrer-Cuzick and Gail breast cancer risk models in a prospective UK screening cohort. Breast Cancer Res..

[30] Vilmun BM (2020). Impact of adding breast density to breast cancer risk models: a systematic review. Eur. J. Radiol..

[31] Hersch J, Jansen J, McCaffery K (2018). Decision-making about mammographic screening: pursuing informed choice. Climacteric.

[32] Schwartz LM, Woloshin S, Fowler FJ, Welch HG (2004). Enthusiasm for cancer screening in the United States. JAMA.

[33] Hoffmann TC, Del, Mar C (2015). Patients’ expectations of the benefits and harms of treatments, screening, and tests: a systematic review. JAMA Intern. Med..

[34] Salzburg Global Seminar. Salzburg statement on shared decision making. *BMJ* **342**, d1745 (2011). 10.1136/bmj.d1745 21427038

[35] Keating NL, Pace LE (2018). Breast cancer screening in 2018: time for shared decision making. JAMA.

[36] Martinez-Alonso M, et al. Assessment of the effects of decision aids about breast cancer screening: A systematic review and meta-analysis. *BMJ Open* **7**, e016894 (2017). 10.1136/bmjopen-2017-016894 PMC5640065 28988175

[37] Hoffman AS (2013). Delivering patient decision aids on the Internet: definitions, theories, current evidence, and emerging research areas. BMC Med. Inform. Decis. Mak..

[38] Health AIo, Welfare. BreastScreen Australia Monitoring Report 2022. AIHW, Canberra (2022).

[39] Tomko C (2015). Decisional outcomes following use of an interactive web-based decision aid for prostate cancer screening. Transl. Behav. Med..

[40] Volk RJ (2007). Trials of decision aids for prostate cancer screening. Am. J. Preventive Med..

[41] Wakefield CE (2008). A randomized controlled trial of a decision aid for women considering genetic testing for breast and ovarian cancer risk. Breast Cancer Res. Treat..

[42] Wakefield CE (2008). A randomized trial of a breast/ovarian cancer genetic testing decision aid used as a communication aid during genetic counseling. Psychooncology.

[43] Peate M (2011). It’s now or never: fertility-related knowledge, decision-making preferences, and treatment intentions in young women with breast cancer—an Australian fertility decision aid collaborative group study. J. Clin. Oncol..

[44] Peate M (2012). Making hard choices easier: a prospective, multicentre study to assess the efficacy of a fertility-related decision aid in young women with early-stage breast cancer. Br. J. Cancer.

[45] Mathieu E (2010). Helping women make choices about mammography screening: an online randomized trial of a decision aid for 40-year-old women. Patient Educ. Counseling..

[46] Mathieu E (2007). Informed choice in mammography screening: a randomized trial of a decision aid for 70-year-old women. Arch. Intern. Med..

[47] Green MJ (2004). Effect of a computer-based decision aid on knowledge, perceptions, and intentions about genetic testing for breast cancer susceptibility: a randomized controlled trial. JAMA.

[48] Stacey D (2017). Decision aids for people facing health treatment or screening decisions. Cochrane Database Syst. Rev..

[49] Ghanouni A (2020). Attitudes towards risk-stratified breast cancer screening among women in England: a cross-sectional survey. J. Med. Screen..

[50] Kelley-Jones C, Scott S, Waller J (2021). UK women’s views of the concepts of personalised breast cancer risk assessment and risk-stratified breast screening: a qualitative interview study. Cancers.

[51] Dunlop K (2021). Acceptability of risk-stratified population screening across cancer types: qualitative interviews with the Australian public. Health Expectations..

[52] Rainey L, van der Waal D, Broeders MJM (2020). Dutch women’s intended participation in a risk-based breast cancer screening and prevention programme: a survey study identifying preferences, facilitators and barriers. BMC Cancer.

[53] Elwyn G (2009). Assessing the quality of decision support technologies using the International Patient Decision Aid Standards instrument (IPDASi). PloS ONE.

[54] Rimer BK, Briss PA, Zeller PK, Chan EC, Woolf SH (2004). Informed decision making: what is its role in cancer screening?. Cancer.

[55] Moser RP, Mccaul K, Peters E, Nelson W, Marcus SE (2007). Associations of perceived risk and worry with cancer health-protective actions:data from the Health Information National Trends Survey (HINTS). J. Health Psychol..

[56] King MT (2018). Australian utility weights for the EORTC QLU-C10D, a multi-attribute utility instrument derived from the cancer-specific quality of life questionnaire, EORTC QLQ-C30. PharmacoEconomics.

[57] Miu AC, Heilman RM, Houser D (2008). Anxiety impairs decision-making: psychophysiological evidence from an Iowa Gambling Task. Biol. Psychol..

[58] Johansson M, Brodersen J (2015). Informed choice in screening needs more than information. Lancet.

[59] Gurmankin Levy A, Shea J, Williams SV, Quistberg A, Armstrong K (2006). Measuring perceptions of breast cancer risk. Cancer Epidemiol. Biomark. Prev..

[60] Glanz, K., Rimer, B. K. & Viswanath, K. *Health Behavior and Health Education: Theory,**Research, and Practice* (Wiley, 2008).

[61] Michie S, Dormandy E, Marteau TM (2002). The multi-dimensional measure of informed choice: a validation study. Patient Educ. Counseling..

[62] Briss P (2004). Promoting informed decisions about cancer screening in communities and healthcare systems. Am. J. Prev. Med..

[63] Dormandy E, Michie S, Hooper R, Marteau TM (2006). Informed choice in antenatal Down syndrome screening: a cluster-randomised trial of combined versus separate visit testing. Patient Educ. Counseling..

[64] O’Connor AM (1995). Validation of a decisional conflict scale. Med Decis. Mak..

[65] Holmes-Rovner M (1996). Patient satisfaction with health care decisions: the satisfaction with decision scale. Med Decis. Mak..

[66] Harris PA (2009). Research Electronic Data Capture (REDCap)—A metadata-driven methodology and workflow process for providing translational research informatics support. J. Biomed. Inform..

[67] Harris PA (2019). The REDCap consortium: building an international community of software platform partners. J. Biomed. Inform..

