# Supplementary material for: Impact of a risk based breast screening decision aid on understanding, acceptance and decision making
Source: NPJ Breast Cancer. 2023 Aug 8;9:65. doi: 10.1038/s41523-023-00569-4 (PMC10409718; doi:10.1038/s41523-023-00569-4)
Supplement: Supplementary file 1 — Supplementary table 1 [file 41523_2023_569_MOESM1_ESM.docx]

# Supplementary Table 1 - Missing values summary

A summary of missing values from the various outputs and survey questions.

| **Demographics** | **Completed** | **Missing** | **Total** |
| --- | --- | --- | --- |
| Age | 242 | 0 | 242 |
| Relationship status | 241 | 1 | 242 |
| Children | 241 | 1 | 242 |
| Do you have daughters? | 210 | 0 | 210 |
| Postcode | 242 | 0 | 242 |
| Education level | 235 | 7 | 242 |
| Employment status | 235 | 7 | 242 |
| Language spoken at home | 242 | 0 | 242 |
| Number of prior mammograms | 241 | 1 | 242 |
| Length of time since last mammogram | 218 | 6 | 224 |
| Prior biopsy | 241 | 1 | 242 |
| Prior breast cancer | 241 | 1 | 242 |
| Prior ovarian cancer | 239 | 3 | 242 |
| Family history | 240 | 2 | 242 |
| Genetic testing | 242 | 0 | 242 |
|  | | | |
| **Pre questionnaire** | **Completed** | **Missing** | **Total** |
| Breast cancer risk factor knowledge subjective | 234 | 8 | 242 |
| Knowledge on personalised breast screening subjective | 233 | 9 | 242 |
| Risk factor knowledge/8 | 241 | 1 | 242 |
| Risk perception/100 | 223 | 19 | 242 |
| Risk perception as a scale | 240 | 2 | 242 |
| Risk perception relative to age matched women | 241 | 1 | 242 |
| OASIS questions | 241 | 1 | 242 |
| Breast cancer worry | 238 | 4 | 242 |
| Personalised breast screening interest | 241 | 1 | 242 |
| Would take part in risk stratified screening? | 241 | 1 | 242 |
| Risk assessment interest | 241 | 1 | 242 |
| Genetic testing interest | 239 | 3 | 242 |
| 3 yearly scenario | 239 | 3 | 242 |
| 5 yearly scenario | 239 | 3 | 242 |
| Annual scenario | 235 | 7 | 242 |
| Risk based screening interest based on change of frequency | 241 | 1 | 242 |
|  | | | |
| **Post questionnaire** | **Completed** | **Missing** | **Total** |
| Breast cancer risk factor knowledge subjective | 123 | 4 | 127 |
| Knowledge on personalised breast screening subjective | 125 | 2 | 127 |
| Risk factor knowledge/8 | 126 | 1 | 127 |
| Risk perception/100 | 122 | 5 | 127 |
| Risk perception as a scale | 127 | 0 | 127 |
| Risk perception relative to age matched women | 127 | 0 | 127 |
| Personalised breast screening interest | 127 | 0 | 127 |
| Preference in individualised screening over current model | 126 | 1 | 127 |
| Risk assessment interest | 124 | 3 | 127 |
| Genetic testing interest | 123 | 4 | 127 |
| 3 yearly scenario | 127 | 0 | 127 |
| 5 yearly scenario | 127 | 0 | 127 |
| Annual scenario | 127 | 0 | 127 |
| Risk based screening interest based on change of frequency | 107 | 20 | 127 |
| Values assessment | 126 | 1 | 127 |
| Preference between 2 choices | 99 | 28 | 127 |
| Satisfaction with decision | 125 | 2 | 127 |
|  | | | |
| **Website feedback** | **Completed** | **Missing** | **Total** |
| Length of time on website | 125 | 2 | 127 |
| Clarity of information | 125 | 2 | 127 |
| Amount of new information | 125 | 2 | 127 |
| Length of website feedback | 126 | 1 | 127 |
| Ease of understanding | 125 | 2 | 127 |
| Ease of navigation | 124 | 3 | 127 |
| Website links clicked? | 124 | 3 | 127 |
| Extra links helpful? | 55 | 0 | 55 |
| Medical definitions used? | 126 | 1 | 127 |
| References clicked? | 126 | 1 | 127 |
| Personal breast cancer risk explanation | 125 | 2 | 127 |
| Help in deciding if individualised screening was right for you | 125 | 2 | 127 |
| Anxious or worried? | 113 | 14 | 127 |
| Anything confusing? | 124 | 3 | 127 |
| Specific phrases make you worried or fearful | 124 | 3 | 127 |
| Suggestions on improvement? | 72 | 55 | 127 |
